# Unveiling four decades of intensifying precipitation from tropical cyclones using satellite measurements

**DOI:** 10.1038/s41598-022-17640-y

**Published:** 2022-08-09

**Authors:** Eric J. Shearer, Vesta Afzali Gorooh, Phu Nguyen, Kuo-Lin Hsu, Soroosh Sorooshian

**Affiliations:** 1 Center for Hydrometeorology and Remote Sensing (CHRS), Department of Civil and Environmental Engineering, University of California, Irvine, Irvine, CA USA; 2 Department of Earth System Science, University of California, Irvine, Irvine, CA USA

**Keywords:** Natural hazards, Hydrology, Attribution

## Abstract

Increases in precipitation rates and volumes from tropical cyclones (TCs) caused by anthropogenic warming are predicted by climate modeling studies and have been identified in several high intensity storms occurring over the last half decade. However, it has been difficult to detect historical trends in TC precipitation at time scales long enough to overcome natural climate variability because of limitations in existing precipitation observations. We introduce an experimental global high-resolution climate data record of precipitation produced using infrared satellite imagery and corrected at the monthly scale by a gauge-derived product that shows generally good performance during two hurricane case studies but estimates higher mean precipitation rates in the tropics than the evaluation datasets. General increases in mean and extreme rainfall rates during the study period of 1980–2019 are identified, culminating in a 12–18%/40-year increase in global rainfall rates. Overall, all basins have experienced intensification in precipitation rates. Increases in rainfall rates have boosted the mean precipitation volume of global TCs by 7–15% over 40 years, with the starkest rises seen in the North Atlantic, South Indian, and South Pacific basins (maximum 59–64% over 40 years). In terms of inland rainfall totals, year-by-year trends are generally positive due to increasing TC frequency, slower decay over land, and more intense rainfall, with an alarming increase of 81–85% seen from the strongest global TCs. As the global trend in precipitation rates follows expectations from warming sea surface temperatures (11.1%/°C), we hypothesize that the observed trends could be a result of anthropogenic warming creating greater concentrations of water vapor in the atmosphere, though retrospective studies of TC dynamics over the period are needed to confirm.

## Introduction

Tropical cyclones (TCs) are among the most devastating and deadly climate phenomena observable globally^1,2^. Extreme precipitation produced by TCs, especially when coupled with wind-enhanced storm surge, culminate to produce some of the most significant flooding damages every year^3–7^. For example, Hurricane Harvey dropped an unprecedented 1.5 m of rainfall over the greater Houston metropolitan region^8,9^ while Typhoon Haiyan caused a catastrophic emergency in the Philippines due to the combination of high storm surge and extreme rainfall totals^10^.

Future climate projections ubiquitously predict increased TC precipitation into the year 2100^11–20^. The intensification of precipitation by anthropogenic warming has been observed in some of the most intense and recent TCs in the North Atlantic basin, namely Hurricane Katrina, Irma, Maria^18^, Harvey^8,21^, and Florence^22^. Regional increases in TC activity have been detected in North America^23–25^, South and Southeast Asia^26–28^, and Australia^29^. However, (1) despite the apparent intensification of TCs in the last few years, (2) the consistent projection of increased TC precipitation by global and regional climate models, and (3) the proven anthropogenic influence on TC precipitation in select regions and case studies, the hypothesis that there has been a historical change in global TC precipitation activity has not reached a consensus in the global community owing to the large natural variability characteristic of TC events^30–33^ and limited observations in the early satellite age.

One of the barriers to making a conclusive assessment of historic TC precipitation is the lack of high-resolution and global precipitation data available for durations long enough to overcome natural climate variability. Thanks to the rapid advancement of satellite precipitation estimating algorithms^34–37^, the first High-Resolution Precipitation Climate Data Records (HRPCDR) are being produced, making precipitation measurements at 0.04° (~ 4 km) and sub-daily spatiotemporal resolution available back to 1980. Available at higher resolutions than other long duration records of precipitation like Precipitation Estimates from Remotely Sensed Information using Artificial Neural Networks-CDR (PERSIANN-CDR^38^) and Climate Hazards Group InfraRed Precipitation with Station data (CHIRPS), HRPCDRs are shown to better capture the pattern and intensity of the most intense precipitation rates^39^—an invaluable improvement to accurately assess TC precipitation.

In this study, a statistical analysis technique known as object-oriented analysis is used to data mine TC precipitation from an HRPCDR produced from PERSIANN Dynamic Infrared Rain rate model (PDIR^40,41^) over the 1980–2019 period. Precipitation measurements within 500 km of TC track data are captured and used to calculate characteristics that describe the event’s hydrometeorological characteristics, namely the mean, 90th, and 99th percentile of precipitation rates, along with the total volume of precipitation and its component over land. The method of using characteristics to describe an event or “object” is known as object-oriented analysis, described in Sellars et al.^42^ and used in Sellars et al.^43,44^, Shearer et al.^45^, and Sadeghi et al.^46^. Characteristics are considered at an annual basis (calendar year) and are calculated per TC basin determined by genesis location: East Pacific (EP), North Atlantic (NA), North Indian (NI), South Indian (SI), South Pacific (SP), and West Pacific (WP), and together as a global aggregate. Trends are further divided into intensity categories based on their peak windfall measurements according to the Saffir-Simpson scale^47^: “All TCs” constitute all events, “Weak TCs” are all sub-hurricane strength storms, “Strong TCs” include category 1–2 hurricanes, and “Very Strong TCs” indicate any storm at or above category 3 (Fig. S1). These results paint a varied history in the last 40 years of TC activity, with notable increases in TC precipitation rates across basins and mixed but generally positive changes in total and inland precipitation volumes between basins.

## Results

**Figure 1:**
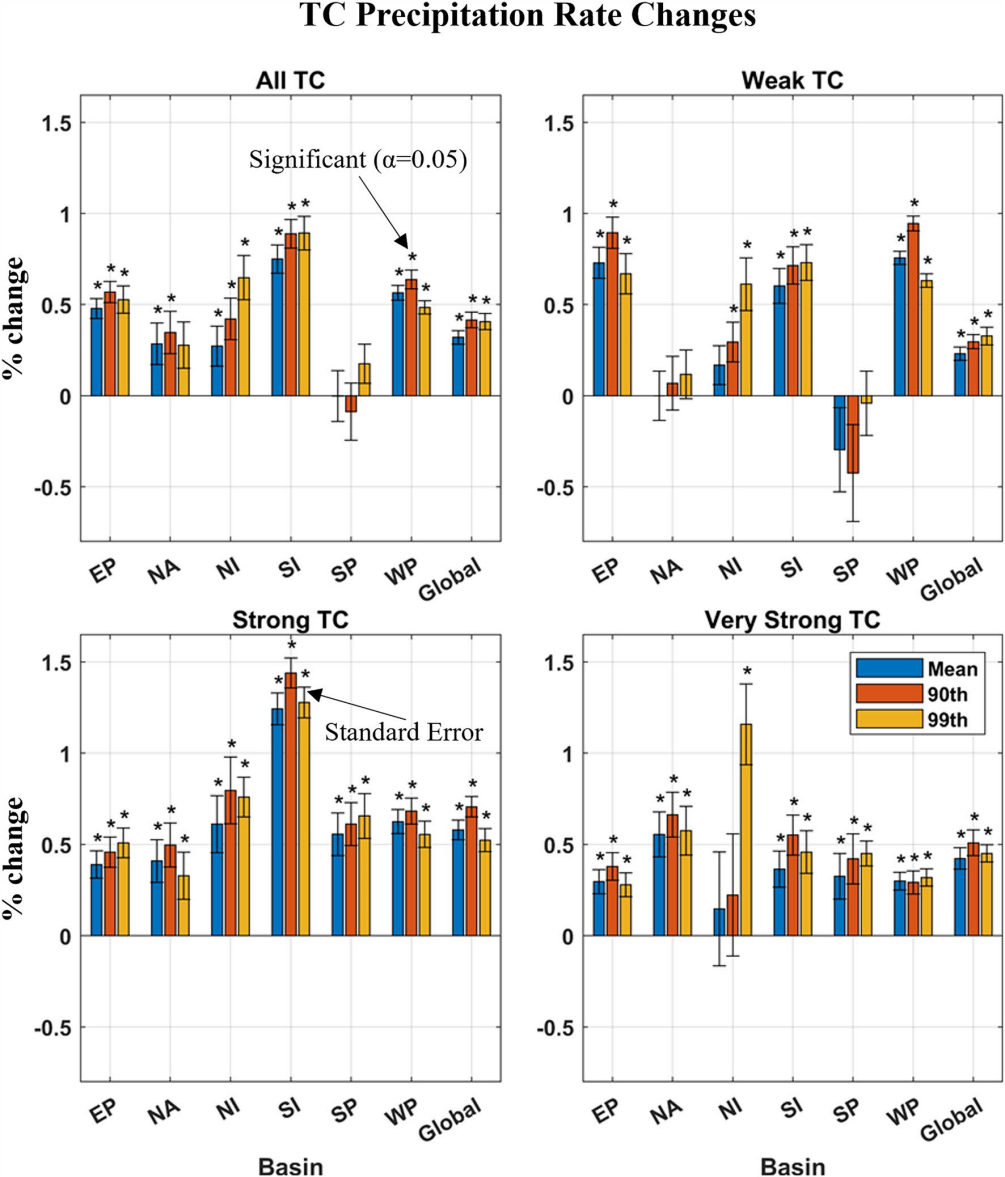
Annual precipitation rate changes of mean and upper percentile precipitation rates by intensity classifications and basins— East Pacific (EP), North Atlantic (NA), North Indian (NI), South Indian (SI), South Pacific (SP), and West Pacific (WP). Rates are calculated from the average year-to-year increase of the fitted linear model. Asterisks represent statistical significance at α = 0.05. During this period, global mean sea surface temperature increased at a rate of 0.13 °C/decade^58^. Knutson et al.^19^ identifies warming in the tropics occurs at ~ 75% the rate of global temperatures, translating into a ~ 0.10 °C/decade trend in the tropics.

**Figure 2:**
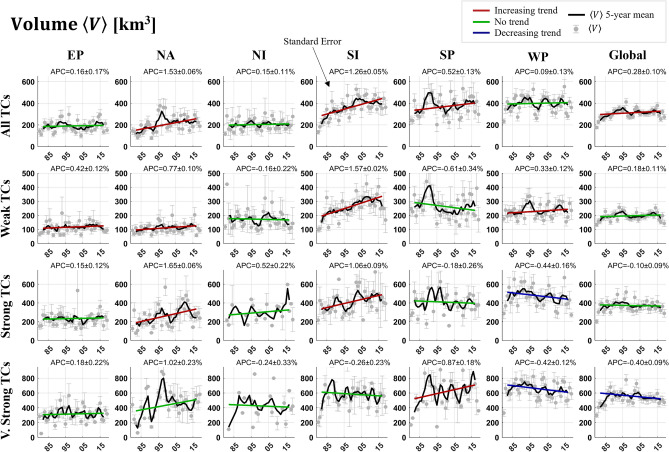
Trend analysis of mean TC precipitation volume totals ($\langle V\rangle$), separated by intensity and basin classifications. Gray values with standard error uncertainty bounds indicate annual average values while black lines display five-year average timeseries data. Colored lines indicate trend of the fitted linear model and the colors red, blue, and green indicate a statistically significant increasing, decreasing, and insignificant trend at α = 0.05, respectively. Numbers on the top right of each subfigure are the annual percent change (APC) of the trend line and the standard error. Time-axis units are the last two digits of the calendar year, from 1980 to 2019.

**Figure 3:**
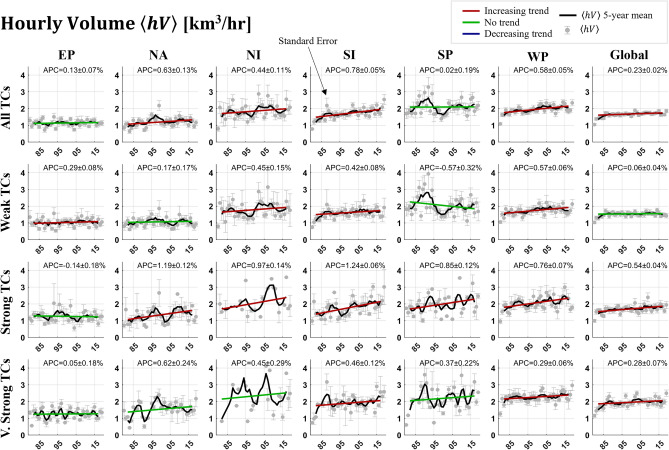
Trend analysis of mean hourly TC precipitation volume totals ($\langle hV\rangle$), separated by intensity and basin classifications. The symbology and features are identical to those from Fig. 2.

**Figure 4:**
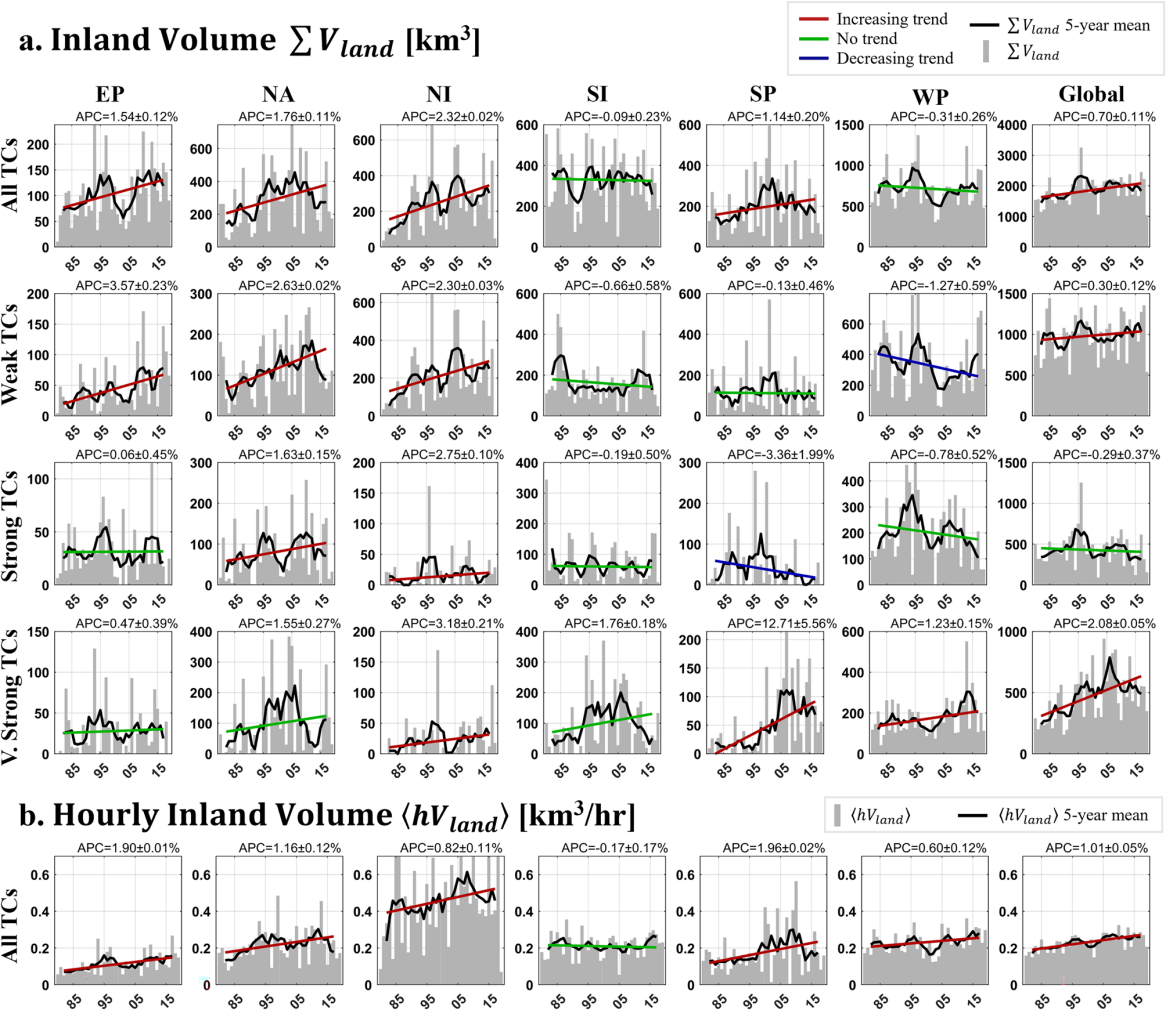
Trend analysis of landfalling tropical cyclone (TC) precipitation volume variables, separated by intensity and basin classifications. Symbology is mostly identical to Figs. 2 and 3, except for gray bars indicating one-year accumulations/averages of landfalling TC precipitation. a. Trends in yearly over land tropical cyclone precipitation volume accumulations ($\sum {V}_{land}$). b. Trends in mean hourly landfalling precipitation volumes ($\langle h{V}_{land}\rangle$), defined as the amount of precipitation an average TC per category drops over land per hour. Note only the All TCs category is shown as values were similar across all intensity categories, making their differences insignificant. Plainly, because $\langle h{V}_{land}\rangle$ from a Weak TC, a Strong TC, and a Very Strong TC are similar, trends are sufficiently represented in the All TCs category.

Recent evidence has suggested that an increase in global TC precipitation rates is detectable in historical rain data archives^48^, though these studies are limited in spatiotemporal extent. To investigate if these trends exist in the climate scale or are rather the result of natural variability in sub-climate timescales, the mean and extreme values of TC precipitation are considered at a yearly basis over a 40-year period. We ask the question: “When considering the distribution of rainfall rate values, do we observe an increase in the mean consistent with expectations of Clausius-Clapeyron to super Clausius-Clapeyron scaling—expected in kilometer- and sub-daily scale processes^49^—and do we see an elongation of the tail indicating greater extreme values?” To probe this question, the mean ($\langle R\rangle$), 90th (${R}_{90}$), and 99th (${R}_{99}$) percentile of precipitation rates over the life cycle of a TC (rainy grid cells only) were calculated, organized by basin and intensity classifications, then averaged annually. $\langle R\rangle$ values range from 1.6 to 2.9 mm/hr, ${R}_{90}$ values range from 4.0 to 8.4 mm/hr, and ${R}_{99}$ values range from 17.2 to 26.9 mm/hr, with the strongest rainfall found in the SP and the weakest in the EP (Fig. S2). To overcome the fundamental variability in TC events, values are smoothed over a five-year window and trends were tested using robust linear fitting, which helps limit the influence of outliers. The results of this analysis can be seen in Fig. 1, where trends in precipitation rates are reported as annual percent changes (APC) measured by the fitted model. In general, most regions, categories, and precipitation rates are seeing significantly increasing trends over the study period. These are reflected by significant increases globally across intensity basins (12–18%/40 years), with average APC in $\langle R\rangle$, ${R}_{90}$, and ${R}_{99}$ of 0.32 ± 0.03%/year, 0.42 ± 0.04%/year and 0.41 ± 0.04%/year, respectively, in the All TCs category and between 0.42 and 0.70%/year (17–28%/40 years) in Strong and Very Strong TCs. However, when considering the “global” category, it is important to note that WP basin has > 30% of global TC occurrences of all basins (Table 1) and will inevitably influence trends greater than other basins—for example the NI basin, which includes less than 10% of TC events per year. This influence will also exist in the results presented in Figs. 2, 3, and 4.

**Table 1:** Tropical cyclone (TC) occurrences from 1980–2019 by basin and intensity. Percentages are per intensity classification (i.e. all percentages in the All Tropical Cyclone (ATC) category add to 100%) and the numbers in parenthesis are the totals. WTC, STC, and VSTC stand for Weak, Strong, and Very Strong TCs, respectively.

	EP	NA	NI	SI	SP	WP
ATC	19% (827)	14% (618)	7% (311)	17% (745)	11% (474)	31% (1,340)
WTC	18% (433)	14% (356)	10% (249)	17% (427)	12% (287)	29% (712)
STC	21% (260)	16% (197)	3% (42)	18% (215)	11% (140)	30% (364)
VSTC	21% (134)	10% (65)	3% (20)	17% (108)	8% (54)	41% (265)

Given the consistent increases in precipitation rates, one may assume that that the mean volume of precipitated water per event has also increased. To determine this, TC volume is measured per storm and averaged yearly. TC volumes are calculated by multiplying each rainy grid cell by the grid cell area (latitude-dependent), then summing over the storm’s lifetime (see the Methods section for specifics on grid size and TC extent). The time series of mean annual TC precipitation volumes ($\langle V\rangle$, hereafter referred to as “volume(s)”) are plotted in Fig. 2, separated by category and basin. Again, five-year smoothing was applied to the time series and trends were tested using robust linear fitting. The time series at the top-right of Fig. 2 provide a summarizing view of global $\langle V\rangle$ trends: increasing at a rate of 0.28 ± 0.10% per year or 7–15% over 40 years, though not without a strong oscillation in the time series. Indeed, most categories have either seen an increasing trend or no trend, though no basin has seen increasing trends across all intensity categories. Of particular interest are the following results: (1) A sharp increase in volumes in the SI (1.26 ± 0.05%/yr or 48–52%/40-yr) and NA (1.53 ± 0.06%/yr or 59–64%/40-yr) basins driven by Weak and Strong TCs; (2) A modest increase in volumes from Very Strong TCs in the SP basin (APC = 0.87 ± 0.18%); and (3) A negative trend in the volumes of Strong and Very Strong TCs in the WP basin of − 0.44 ± 0.16%/yr and − 0.42 ± 0.12%/yr, respectively, the latter of which has seemingly caused a 40-year decrease in global Very Strong TC volumes of 12–20%. Overall, Weak TCs have seen increases in four of six basins, Strong TCs have changed in three of six, with two positive trends and the other negative, and Very Strong TCs have an increasing and decreasing trend in one basin each. This culminates into the All TCs category having increased in three out of six basins.

Trends in $\langle V\rangle$ could be interpreted as being a result of increases in TC duration and not necessarily precipitation intensity. Increases in global SST values have been linked to slowing of TC translation speeds^50^ and slower decay following landfall^51^ both increasing TC duration values. We normalize the $\langle V\rangle$ quantity by its duration in hours to produce hourly $\langle V\rangle$, hereafter symbolized as $\langle hV\rangle$. The results of this analysis, shown in Fig. 3, is that across categories there have only been increasing or non-significant (no) trends. Only one basin/intensity pair has a no trend in $\langle hV\rangle$ and a positive trend in $\langle V\rangle$, which indicates an increase in volume from an increase in duration rather than changes in precipitation. All other time series with positive trends in $\langle V\rangle$ have also experienced an increase in $\langle hV\rangle$ (max: 1.24% ± 0.06 per year or 52% over the 40-year period). Simply put, in most cases an increase in TC precipitation volume is at least partially a result of increasing precipitation rates and not solely duration. Increasing trends in Very Strong TC $\langle hV\rangle$ in the global and WP categories hint that the corresponding decreasing trends in $\langle V\rangle$ are a result of a decrease in event duration. Further analysis into TC durations confirms these results, with decreasing trends of − 0.56 ± 0.11% and − 0.32 ± 0.09% for WP and global Very Strong TC duration, respectively. Other notable negative trends in duration (Table 2) occur in global Strong TCs (− 0.51 ± 0.08%/year) and All TCs in the NI (− 0.43 ± 0.16%/year) and WP (− 0.44 ± 0.12%/year) basins, while increasing trends occur in NA (0.78 ± 0.07%/year) and SI (0.53 ± 0.08%/year). These results are calculated solely from track data. In general, these results are in line with the analysis done in Lavender and McBride^52^, who found that 70% of variance in the trends of TC precipitation volume during the 1998–2014 period could be explained by changes in TC duration.

**Table 2:** The annual percent change in TC duration over the 1980–2019 period. Bolded numbers indicate statistically significant trends at α = 0.05.

	EP	NA	NI	SI	SP	WP	Global
ATC	0.28 + 0.13%	**0.78 + 0.07%**	**− 0.43 ± 0.16%**	**0.53 + 0.08%**	0.12 ± 0.14%	**− 0.44 ± 0.12%**	**− **0.00 ± 0.08%
WTC	**0.66 ± 0.08%**	**0.71 ± 0.07%**	**− 0.87 ± 0.19%**	**0.86 ± 0.04%**	**− **0.30 ± 0.19%	**− **0.29 ± 0.16%	0.02 ± 0.08%
STC	0.30 ± 0.13%	0.21 ± 0.12%	0.03 ± 0.39%	**− 0.32 ± 0.07%**	**− 0.79 + 0.33%**	**− 1.23 ± 0.14%**	**− 0.51 ± 0.08%**
VSTC	0.07 ± 0.16%	0.46 ± 0.20%	**− **0.03 ± 0.14%	**− 0.46 ± 0.16%**	**0.60 ± 0.14%**	**− 0.56 ± 0.11%**	**− 0.32 ± 0.09%**

Considering the observed increases in rainfall rates and volumes, we now consider if changes are occurring over human population centers, i.e. do we see greater TC precipitation volumes over land? To calculate this metric ($\sum {V}_{land}$, where *land* indicates the landfalling component of TC volume and the summation sign indicates a yearly accumulation rather than an average), precipitation volumes are decomposed into their fractions over oceans and land and accumulated annually. As can be seen in Fig. 4a, increasing trends are the most common of all trends, where they are recorded in 16 out of 28 (57%) basins, while only Weak TCs in the WP and Strong TCs in the SP have seen decreasing trends. Most positive trends can be seen in the Very Strong TC category, where they’re recorded in four out of six basins. Global Very Strong TCs have changed at an alarming rate, with a calculated increase of 81–85% over the study period (2.08 ± 0.05%/year). Weak TCs have increased (changed) in three (four) out of six basins, most notably in the NA by 104–106% in 40 years (2.63 ± 0.02%/yr). Overall, these increases are enough for the global trend to be positive (0.30 ± 0.12%/year). Strong TCs have increased in two basins but aren’t enough to produce a significant trend in the global category. Overall, $\sum {V}_{land}$ in All TCs has increased globally by 24–32% in 40 years (0.70 ± 0.11%/year) and in four out of six basins.

As values of $\sum {V}_{land}$ are inevitably linked to the number of landfalling TCs in a year and the duration they remain over land, trends in $\sum {V}_{land}$ were correlated against those of TC duration over land and landfalling frequency (Tables 3 and 4). Consistent with expectations, most categories correlate with landfalling frequency at α = 0.05: this is true in 20 out of 28 (71%) of the time series and 100% of the Very Strong TC timeseries. Likewise, $\sum {V}_{land}$ correlates with duration over land in 17 of 28 (61%) of the time series. Curiously, time series of global All TCs, Weak TCs, and Strong TCs are uncorrelated with TC landfalling frequency.

**Table 3:** Correlation coefficient of $\sum {V}_{land}$ and annual frequency of landfalling TCs. Bolded numbers indicate statistically significant trends at α = 0.05.

	EP	NA	NI	SI	SP	WP	Global
ATC	**0.50**	**0.43**	**0.84**	**0.71**	0.17	**0.44**	**− **0.06
WTC	**0.76**	0.06	**0.90**	0.29	**0.35**	**0.67**	**− **0.23
STC	0.20	0.22	**0.67**	**0.73**	**0.58**	**0.38**	0.13
VSTC	**0.61**	**0.87**	**0.68**	**0.82**	**0.48**	**0.57**	**0.73**

**Table 4:** Correlation coefficient of $\sum {V}_{land}$ and TC duration over land. Bolded numbers indicate statistically significant trends at α = 0.05.

	EP	NA	NI	SI	SP	WP	Global
ATC	**0.60**	**0.85**	**− **0.30	**0.33**	**0.46**	**0.39**	**0.78**
WTC	0.23	**0.67**	**− **0.04	**0.46**	**0.57**	**0.39**	**0.70**
STC	**0.34**	**0.78**	0.24	0.27	**0.43**	**0.56**	**0.79**
VSTC	0.21	0.25	0.24	0.23	0.27	0.07	**0.74**

To investigate how precipitation intensity over land—independent of frequency and duration—has changed over time, a new variable was calculated: hourly mean precipitation volume over land ($\langle h{V}_{land}\rangle$), which in essence is $\sum {V}_{land}$ normalized by its temporal and frequency components. Or it can also be thought of as the over land component of $\langle hV\rangle$. In the simplest terms, it is the average precipitation volume a TC drops over land in one hour. As shown in Fig. 4b, most basins—as well as the global category—have seen an increase in $\langle h{V}_{land}\rangle$ over the period, indicating an increase in precipitation volumes over land independent of frequency or duration, except for in the SI basin where $\sum {V}_{land}$ is also stagnant. Increases are significant, with APC of 1.90 ± 0.01%/year, 1.16 ± 0.12%/year, 1.96 ± 0.02%/year, and 1.01 ± 0.05%/year recorded in the EP, NA, SP, and globally, respectively (max 78–79% over the study period).

**Figure 5:**
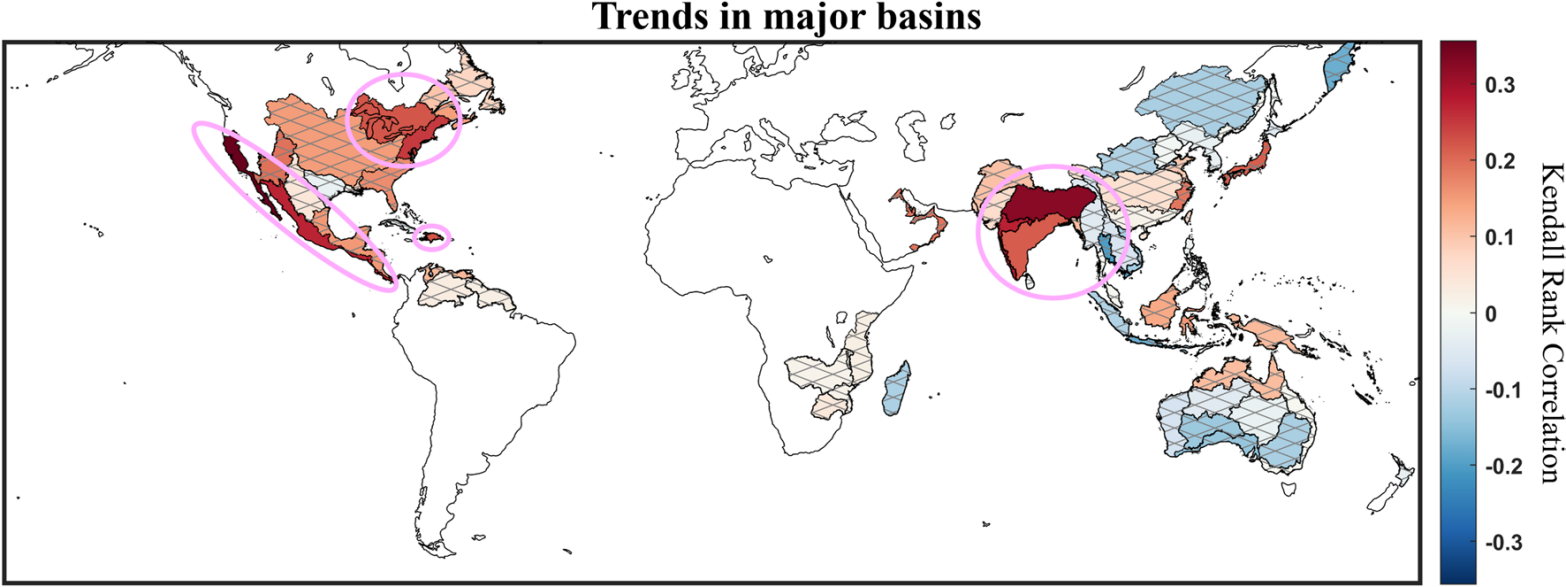
Inland precipitation accumulation trends from 1980–2019 calculated at the major river basin scale. Regions of non-significant trends are indicated by gray hatching. Pink circles denote regions where inland penetration from TCs have been heightened significantly.

The trends in $\sum {V}_{land}$ presented in Fig. 4a can be considered concurrently with Fig. 5, where trends in $\sum {V}_{land}$ are tested spatially over major river basins using the Kendall rank correlation metric (useful for basins for years with no activity; see Materials & Methods for more information)—though only for the “All TCs” category. Basins are only considered if over half the years on record include a nonzero value of $\sum {V}_{land}$, which meant that five-year smoothing could not be used as it was for Figs. 1, 2, 3 and 4. In summary, every major river watershed that recorded significant changes are located within the North American continent and the Indian subcontinent and have witnessed increases in $\sum {V}_{land}$. This helps illustrate the positive trends in $\sum {V}_{land}$ uncovered in Fig. 4 for the NA, EP, and NI basins. East Asia and Australia have seen mixed insignificant trends, consistent with the no trend results for the WP and SI basin, though at odds with the increasing trend found for the SP basin. This can be explained by differences in the trend detection tool. For reference, Fig. S3a,b shows where TC precipitation is located and its contribution to climatology. Between the insignificant trends detected in Fig. 5a and the lack of appreciable rainfall from TCs shown in Fig. S3, the trends in the Arabian Peninsula, East Africa, South America, and the Maritime Continent basins are ignored in this figure and in further discussion.

The variation in trends of TC precipitation is significant: one cannot extrapolate changes in activity in one basin and apply it to another, even for basins close in proximity (example: SI and SP). In order to give an overview of what changes in TC activity have occurred within each basin, we use the following criteria to grade changes of the $\langle R\rangle$, ${R}_{90}$, and ${R}_{99}$, $\langle V\rangle$, and $\sum {V}_{land}$ variables in Table 5:

- Little to no changes: > 50% of the category is not seeing a significant change.
- Mixed changes: ≥ 50% of the category is seeing a significant change, but the trends are mixed between increasing and decreasing.
- Significantly increased: ≥ 50% and < 100% of the category is seeing a significant positive change.
- Very significantly increased: 100% of the category has seen a significant positive change.

**Table 5:**
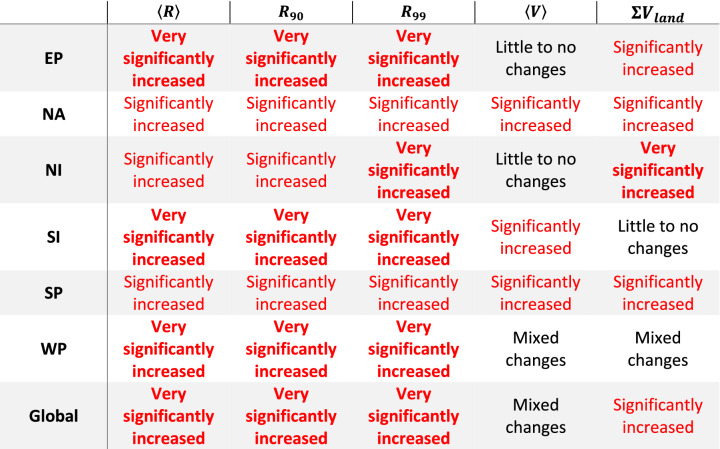
Summary of TC precipitation trends relating to precipitation rate percentile and volume totals across basins and at the global scale. Red text shows trends where at least 2 of 4 intensity classifications have experienced statistically significant positive changes in precipitation and bolded red text indicates categories with positive changes in precipitation in all intensity classifications.

Percentages are calculated based on the number of intensity categories out of four (All, Weak, Strong, and Very Strong) that are seeing a significant trend for each variable. Though not independent of the other categories, the All TCs classification is included in the grading to ensure the Strong and Very Strong categories are not overrepresented against Weak TCs, which are the most frequent TC grade. In most cases, its inclusion does not change results. Note that the $\langle hV\rangle$ and $\langle h{V}_{land}\rangle$ variables are not considered, as they are closely related to TC precipitation rates.

## Discussion

In this study, precipitation volumes and rates from TCs were investigated globally using robust regression tools, where it was shown that pronounced increases in TC precipitation rates can be observed across intensity categories and TC basins. These increases in precipitation rates have created increases in precipitation volumes over many basins and intensity categories, though global results are mixed. In terms of human impacts, greater precipitation totals over land were seen in all basins across categories, many influenced by global increases in TC precipitation along with changes in TC duration or landfalling frequency. Spatially, the most pronounced increases are occurring in North America and the Indian subcontinent. All basins are experiencing intensifying rainfall from TCs in one form or another, indicating changes in the Earth’s hydroclimate over the 1980–2019 period. We hypothesize that observed changes are a result of increases in atmospheric water vapor following Clausius–Clapeyron scaling, with possible contributions by anthropogenic aerosols^53^. However, results from Traxl et al.^54^ caution against using solely thermodynamical results to link detected changes to anthropogenic warming.

Global assessment of TC precipitation trends has been limited due to a lack of appropriate data. Concretely, Section 8.3.2.5 in the 2021 IPCC Working Group 1 summary^20^ reports a “low confidence” that there has been an increase in global TC precipitation, citing limitations in historical observations. Though not without uncertainty, the HRPCDRs methodology explored in this study translates historic satellite observations into data with global (land and water) coverage, a long and homogeneous temporal extent, and high-spatiotemporal resolution. This contrasts with studies that rely on gauge or radar-gauge data^28,55,56^ that are the highest-quality measurements readily available for precipitation-oriented research but are limited by a lack of coverage over the oceans and in sparsely populated regions. Likewise, many studies that rely on satellite measurements are too limited in duration to overcome decade-scale climate oscillations that affect TC activity^48,57^. Moreover, high-resolution precipitation data sets like Tropical Rainfall Measuring Mission (TRMM) Multi-satellite Precipitation Analysis (TMPA) (pre-version 7) and Integrated Multi-satellitE Retrievals for GPM (IMERG) are also not developed to be homogeneous in extended time ranges. Current homogeneous satellite-based CDRs and reanalysis products available for long durations like PERSIANN-CDR or CHIRPS are available at spatiotemporal resolutions too coarse to resolve the topographically complex patterns of the TC cloud shield and movement of the mesoscale phenomenon. At the same time, this study is limited by using a static shape and size threshold to truncate TCs and by its reliance on a new and largely untested dataset that is still coarser in temporal resolution than the scale of TC dynamics.

**Figure 6:**
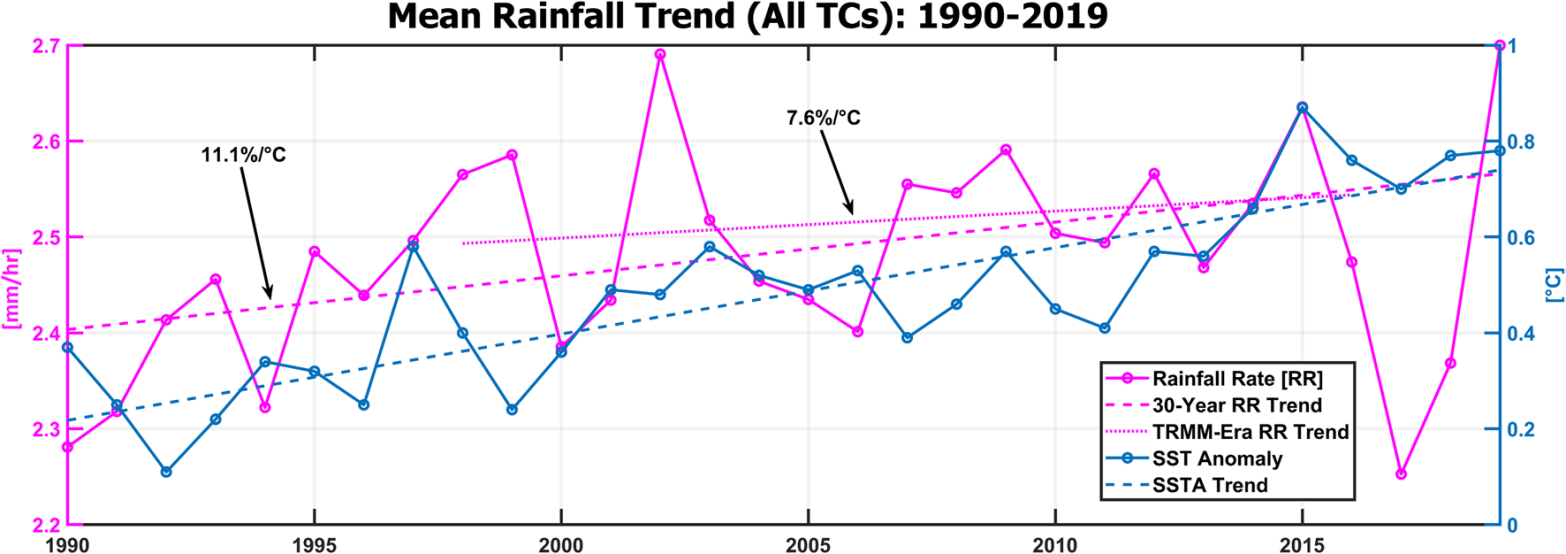
Mean precipitation rate values from All TCs (no smoothing) measured by PDIR-CDR during the 1990–2019 period (magenta) compared to sea surface temperature anomaly measurements (blue) from NOAA NCEI’s Climate at a Glance data portal^58^. Two trends are analyzed: 30-year (1990–2019) and TRMM-era (1998–2016), which show an increase in precipitation rates of 11.1%/C and 7.6%/C, respectively.

Recently, a comparable study by Guzman & Jiang^48^ that was conducted using similar methods to this study and TMPA rainfall data uncovered an increasing trend of 1.3% a year in mean TC precipitation rates and attributed it to increases in global mean temperature. This is significantly higher than the results from Fig. 1, where global TC precipitation rates are shown to be increasing at a rate of 0.32 ± 0.04% a year. Guzman & Jiang’s results suggests a 21% increase in precipitation rates have occurred between 1998 and 2016. Using global mean sea surface temperature (SST) data from NOAA NCEI’s Climate at a Glance data portal^58^, an increase in global ocean SST of 0.28 °C was recorded (note that Guzman & Jiang record this temperature as 0.21 °C using SST measurements localized to TC tracks—Knutson et al.^19^ reports that tropical SST warms at a rate ~ 75% of global SST, so these numbers are consistent with each other). Guzman & Jiang’s rate of 75%/°C is considerably higher than modeling studies’ suggestion that an increase of 7% to ~ 14% (super Clausius-Clapeyron scaling) should occur per 1 °C of global SST warming^19^. Over the same period (1998–2016), our methodology estimates a 2.1 ± 1.3% (7.6%/°C) increase in precipitation rates (Fig. 6), more in line with modeling projections than the quantities given in the comparative study. Our study suggests that over the last thirty years, an increase of 5.0 ± 1.4% occurred during a warming of 0.45 °C (11.1%/°C), again closer in line with modeling studies. The differences between studies are evident when considering mean precipitation values from each study over this period: the range of recorded yearly average precipitation rates from 1998 to 2016 measured by PDIR-CDR was 2.5–2.6 mm/hr, much larger and in a much smaller range than those recorded by TRMM (specifically, TMPA-3B42 v7) using the 500 km truncation method (~ 1.7–2.4 mm/hr). Over the entire 40-year period, precipitation averages recorded by PDIR-CDR range from 1.8 to 2.6 mm/hr. Clearly, the choice of precipitation data set plays a very significant role in the results recorded between studies.

Continued improvement of HRPCDRs like PDIR-CDR and the passage of time will shed further light on how precipitation in historic TCs has evolved because of anthropogenic influence. At this time, further analysis into how climate oscillations influence the variability in TC precipitation properties, identifying when anthropogenic warming began to affect TC characteristics in longer duration historic data sets, and merging observed historical trends with projected future trends are vital to further understanding the past, present, and future of TC precipitation. As TC-linked disasters are becoming more commonplace, our continued understanding of their nature is vital to ensure global security of our coastlines, population centers, and vital infrastructure.

## Materials and methods

### PDIR-CDR precipitation data

Upon the development of the first HRPCDR^39^, it was shown that one of its distinct advantages over PERSIANN-CDR is in recording extreme precipitation. Given the intensity of rainfall and smaller scale of TCs, especially the sharp gradients of the rainfall field found from the core to the outer extent of a TC, it was shown that an HRPCDR could be a more effective tool for producing a climate-scale catalog of TC precipitation than PERSIANN-CDR. For example, the HRPCDR in Sadeghi et al.^39^ outperforms PERSIANN-CDR as a quantitative precipitation estimating (QPE) method for recording the rainfall of Hurricane Harvey.

For this study, we produce an HRPCDR based on the PDIR satellite precipitation measurement algorithm^40,41^, hereafter referred to as “PDIR-CDR”. PDIR-CDR uses three-hourly and 0.04° spatiotemporal resolution measurements of IR-derived cloud-top temperature from the GridSat-B1 archive, which is a consistent and homogenous remotely sensed data set available near-continuously since 1980. PDIR-CDR’s domain, like many other QPE products, is limited to sub-polar regions (60°S to 60°N.) In the instance of missing coverage—mostly occurring in the 1980–1982 period and in the Eastern hemisphere—precipitation estimates from the NASA Modern-Era Retrospective analysis for Research and Applications, Version 2 (MERRA-2^59^) reanalysis project is downscaled from its native 0.625° × 0.5° spatial resolution and used to fill in the gaps. As the spatial resolution of MERRA is coarser than GridSat-B1, some of the finer spatial patterns in the rain field are lost in the final product, but the inclusion of these years was determined to not seriously change the results of this study. Afterwards, the monthly accumulations of precipitation estimates are bias corrected at the monthly scale using Global Precipitation Climatology Project (GPCP) v2.3 monthly gridded gauge data^60^, following the homogeneity-focused methodology of other PERSIANN-based CDRs^38,39^. Though experimental in nature, continued evaluation of PDIR-Now shows generally skillful performance across temporal and spatial scales^61,62^. For more information on PERSIANN data sets, refer to Nguyen et al.^63,64^.

**Figure 7:**
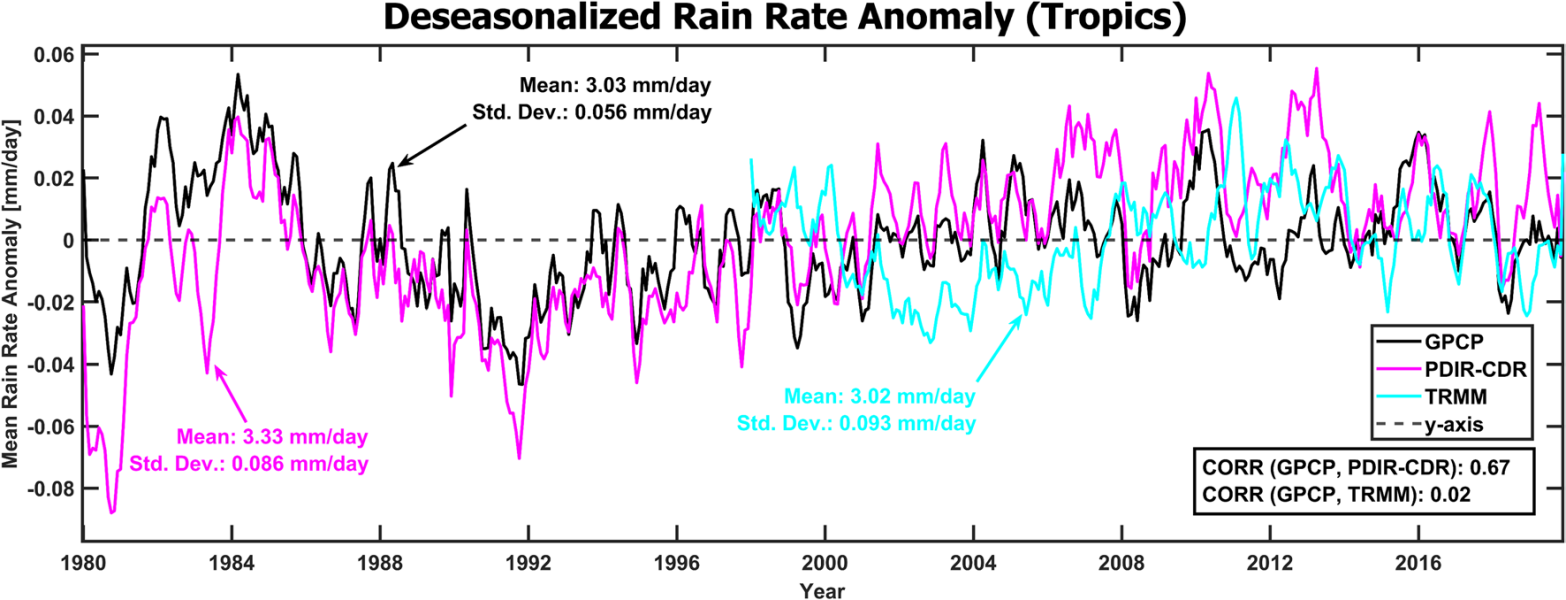
Deseasonalized rain anomalies over the tropics (30°N to 30°S) from GPCP v2.3 (black), PDIR-CDR (magenta), and TRMM TMPA-34BT v7 (cyan), measured at the monthly scale. Each data set is described by its mean value and standard deviation over the domain in the colored arrows. Correlations between PDIR-CDR and TRMM with GPCP are reported in the bottom right box.

One of the most prominent concerns when producing and utilizing high-resolution data sets of precipitation is their uncertainty. Uncertainty is an unavoidable issue when dealing with satellite remote sensing of precipitation at all scales. Even the best multi-input and gauge corrected data sets have considerable issues with accuracy when compared to quality-controlled radar and gauge products^63,64^. This issue is complicated by the fact that there is no perfect ‘ground truth’ data set to compare most products to as the climate is not regularly and directly measured except at a handful of in situ sites^65^ This is especially true in remote regions or over large bodies of water, where satellite and reanalysis are the only options. To address PDIR-CDR’s uncertainty for measuring TC-linked precipitation, we evaluate PDIR-CDR against GPCP v2.3 in the tropics (30°S to 30°N), which shows PDIR-CDR’s strong correlation with GPCP (CORR = 0.67), though not without notable overestimation bias (Fig. 7). Though with notable bias and periods of disagreement with GPCP, primarily in the early record (1980–1984), PDIR-CDR does not show the presence of long-term artificial trends that would indicate inhomogeneity, meaning its weaknesses should not dominate trend analysis results as error is distributed quasi-uniformly across the study period. Moreover, robust regression techniques (summarized later in this section) were used to limit the influence of outliers, including periods of disagreement between PDIR-CDR and GPCP. Overestimation bias is expected from PDIR-CDR because the PDIR algorithm was developed with extreme events in mind, which are frequently underestimated by other QPE techniques^40,41^. PDIR can better capture the upper tail of extreme rainfall at the cost of frequent overestimation. Moreover, the difference in native resolutions between GPCP (2.5°, monthly) and PDIR-CDR (0.04°, 3-hourly) means that short-duration heavy rain events that are captured in PDIR-CDR are smoothed over in GPCP. PDIR-CDR’s high correlation with GPCP is far superior to TRMM’s (TMPA-3B42 v7) (CORR = 0.02), though TRMM shows essentially no bias at the monthly scale.

**Figure 8:**
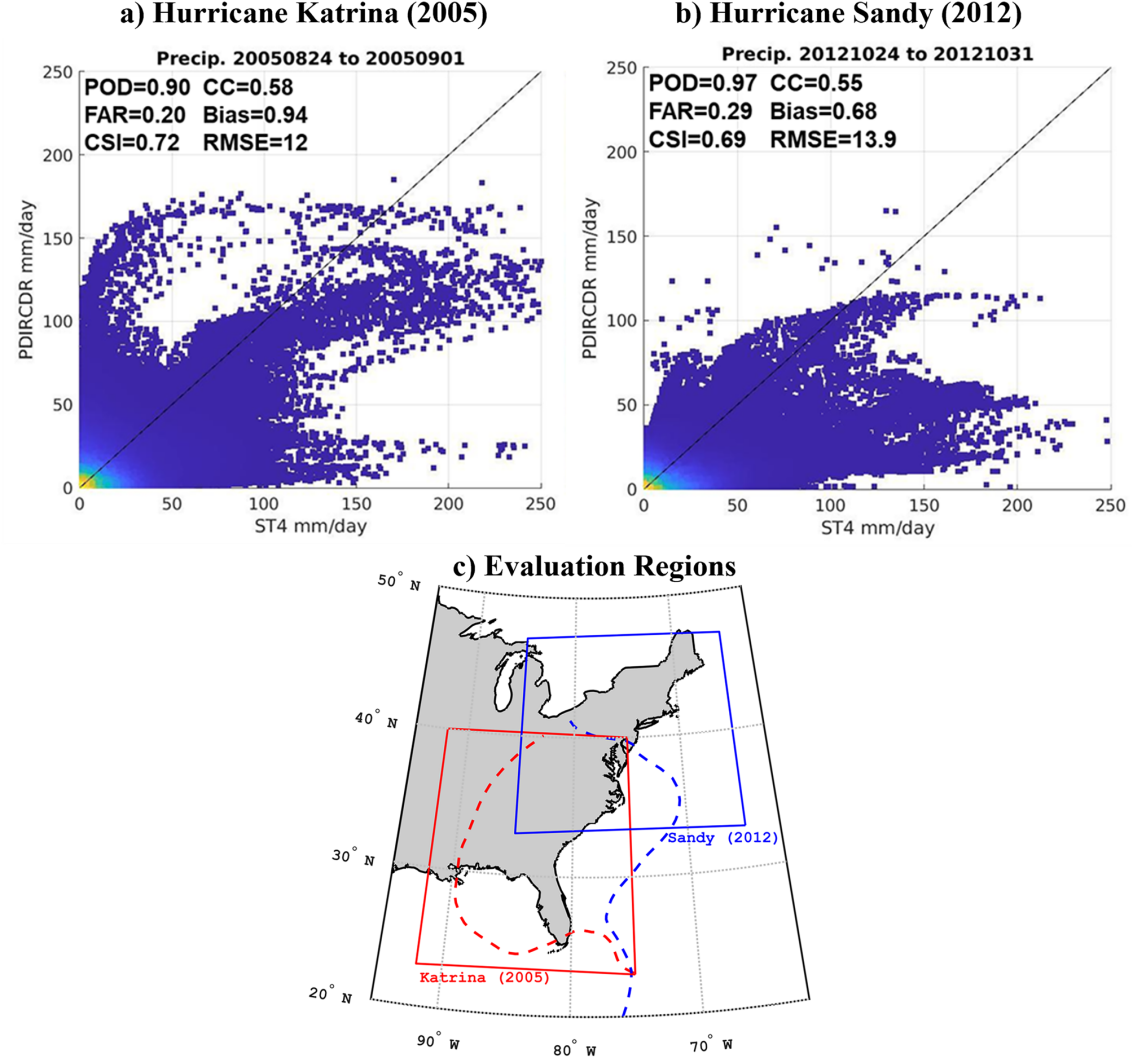
Evaluation of PDIR-CDR rainfall data against Stage IV (ST4) radar-gauge data for (**a**) Hurricane Katrina from Aug-24-2005 to Sep-01-2005 and (**b**) Hurricane Sandy from Oct-24-2012 to Oct-31-2012. Scatter plots of daily pixel totals are shown with Probability of Detection (POD), False Alarm Ratio (FAR), Critical Success Index (CSI), Pearson Correlation Coefficient (CC), Multiplicative Bias, and Root Mean Squared Error (RMSE) scores superimposed. The scatterplot’s color map represents the density of points, with the greatest density at the origin point in yellow. (**c**) The spatial domain (solid line) of each scatter plot with the corresponding hurricane track (dashed line). Hurricane Sandy is shown in blue while Hurricane Katrina is shown in red.

Further analysis of PDIR-CDR performance and uncertainty was done for select case studies to prove its effectiveness for the capture of extreme precipitation, especially over land. In Fig. 8 daily rainfall totals from PDIR-CDR are evaluated in comparison to NOAA’s Stage IV radar-gauge product, a high quality QPE product frequently used for evaluation purposes, during two noteworthy hurricanes that made landfall over large population centers in the United States during the last two decades: Hurricane Katrina (2005) and Hurricane Sandy (2012). By observing the scatter plots of PDIR-CDR and Stage IV comparisons, we see that though there exists a large amount of noise and bands of notable overestimation and underestimation by PDIR-CDR for Hurricane Katrina and Sandy, respectively, the large cluster of points along the perfect correlation line at the extreme end of the chart (> 100 mm/day) show that PDIR-CDR’s quality does not decay for large extreme precipitation totals, an important requirement for this study. For both evaluations, PDIR-CDR’s performance recorded by statistical comparison metrics remained similar: Probability of detection (POD) of or greater than 0.90, false alarm ratio (FAR) of less than 0.30, cumulating into a combined skill index (CSI) score of ~ 0.7, an impressive score when considering satellite QPE at the daily scale. PDIR-CDR’s positive bias of 0.68 and 0.94, combined with notable root mean squared error (RMSE) scores of 12 mm and 13.9 mm, indicate PDIR-CDR’s biggest shortcomings are its tendency to overestimate rainfall, while its FAR scores mean PDIR-CDR regularly records rainfall where there is none. Compare these results to those from Omranian^66^, who found similar performances (CSI of ~ 0.7 and correlation of ~ 0.6) during Hurricane Harvey using what is widely regarded as one of those most accurate satellite remote sensing products, IMERG. Though it’s clear that PDIR-CDR is not the perfect dataset, its high CSI score combined with its notable skill for capturing extreme precipitation totals means its well suited for this study.

### Tropical cyclone segmentation

We use the International Best Tracks Archive for Climate Stewardship (IBTrACS^47^) reanalysis data set for TC track data. IBTRaCS is a collaborative effort to produce TC tracks using reanalysis techniques from climate monitoring centers across the globe. Data availability is three-hourly, corresponding to the resolution of PDIR-CDR. IBTrACS data is used for TC track coordinates from 1980 to 2019 across six basins: EP, NA, NI, SI, SP, and WP. Note that the Southern Atlantic basin was omitted due to sparse activity and the Australian basin was split between the SI and SP basins per IBTrACS convention. Additionally, IBTrACS convention grades TC intensity on the Saffir-Simpson scale regardless of basin.

To designate rainfall as linked to a TC event, a 500-km buffer is drawn around each IBTrACS centroid location: all rainfall within this buffer is recorded as TC rainfall while all remaining rainfall pixels are ignored. The 500-km radius of TC rainfall is based on the physical structure of a fully formed TC and contains the entirety of the precipitation fields for 90% of TC events^67–71^. It should be stated that assuming a perfect circular threshold with a static threshold is a considerable oversimplification of TC structure, which frequently occur in oblong shapes—especially before cyclogenesis—at spatial scales that drastically differ between storms with otherwise similar intensities^72^. However, without readily available data on the radius of the outermost closed isobar, a common way to segment the boundary of a TC—calculated systematically in Weber et al.^73^ but not made publicly available—alternative methods are questionably better than our simplified approach. For example, Skok et al.^56^ uses an object-based approach but fails to detect precipitation in 12% of global TC events. Likewise, the outmost radius of 34-kt winds provided by IBTrACS, another metrics useful for calculating the TC size is not available for all basins (e.g. NI, EP) and is significantly less than true TC size^74^. For these reasons, the simplified approach of the 500-km buffer is preferred for this study, though will result in overestimation bias in the results due to detection of non-TC precipitation signaturess^75^.

### Trend analysis tests

As the natural variability of TC characteristics and the uncertainty of rainfall estimates in the early data period are large, trend analysis was calculated using robust regression^76,77^. M-estimation, a class of extremum estimators, are robust regression techniques that are often used as an alternative to the linear-least squares method due to their decreased sensitivity to outliers and independence from assuming distribution. Robust regression calculations presented in this paper were performed using MATLAB’s “robustfit” function that is based on the iteratively reweighted least squares M-estimation method^78^. Reductions in degrees of freedom caused by five-year smoothing were considered during trend analysis calculations. All robust regression calculations were tested against a *p*-value = 0.05.

The Mann–Kendall test is a non-parametric trend analysis test that analyzes data collected over time for monotonic (consistently increasing or decreasing) trends. The Mann–Kendall test’s non-parametric nature means its suitable for use when data points are missing, making it useful for time series with missing data points, e.g. for analysis of basins with years without precipitation. Based on the Mann–Kendall test, the Kendall rank correlation ($\tau$) is a normalized trend analysis metric with bounds [–1, 1] corresponding to a perfectly increasing/decreasing time series. It is calculated thusly:

$$\tau =\frac{\sum_{i=1}^{ n-1}\sum_{j=i+1}^{ n}sgn\left({x}_{j}-{x}_{i}\right)}{n(n-1)/2}$$

where *x* and *n* represent sample number and population size, respectively.

### Continuous and categorical skill metrics

The performance of PDIR-CDR was evaluated by the following continuous skill metrics: Pearson correlation coefficient (CORR), multiplicative bias (BIAS), and root-mean-square error (RMSE)

$$CORR = \frac{1}{n}\frac{{\mathop \sum \nolimits_{i = 1}^{n} \left( {P_{i} - \overline{P}} \right)\left( {O_{i} - \overline{O}} \right)}}{{\sqrt {\mathop \sum \nolimits_{i = 1}^{n} \left( {P_{i} - \overline{P}} \right)^{2} } \sqrt {\mathop \sum \nolimits_{i = 1}^{n} \left( {O_{i} - \overline{O}} \right)^{2} } }}$$

$$BIAS = \frac{1}{n}\mathop \sum \limits_{i = 1}^{n} \frac{{P_{i} }}{{O_{i} }}$$

$$RMSE = \sqrt {\frac{1}{n}\mathop \sum \limits_{i = 1}^{n} \left( {P_{i} - O_{i} } \right)}$$

where *P*_*i*_ and *O*_*i*_ represent the *i*th precipitation estimate from the model and observation data sets, respectively, and *n* is the number of data comparisons.

We use also evaluate PDIR-CDR using categorical skill metrics: Probability of Detection (POD), False Alarm Ratio (FAR), and Critical Success Index (CSI)

$$POD=\frac{H}{H+M}$$

$$FAR=\frac{F}{F+H}$$

$$CSI=\frac{H}{H+F+M}$$

where *H* (hit) indicates that the model and observation dataset agree on the presence of rainfall, *M* (miss) identifies events detected in observations but missed by the model, and *F* (false alarm) indicates events detected by the model but not confirmed by observation.

## Supplementary Information

Supplementary Information.

## Data Availability

The PDIR-CDR dataset generated and analyzed during the current study is available from the corresponding author on reasonable request.

## References

[1] Rappaport EN (2014). Fatalities in the United States from Atlantic tropical cyclones: New data and interpretation. Bull. Am. Meteor. Soc..

[2] Bakkensen, L. A., & Mendelsohn, R. O. Global tropical cyclone damages and fatalities under climate change: An updated assessment. In *Hurricane Risk* 179–197. (Springer, Cham, 2019). 10.1007/978-3-030-02402-4_9.

[3] Dube SK, Jain I, Rao AD, Murty TS (2009). Storm surge modelling for the Bay of Bengal and Arabian Sea. Nat. Hazards.

[4] Bank, W. The World Bank Annual Report 2010. (2010) 10.1596/978-0-8213-8376-6.

[5] Managing the risks of extreme events and disasters to advance climate change adaptation—IPCC. https://www.ipcc.ch/report/managing-the-risks-of-extreme-events-and-disasters-to-advance-climate-change-adaptation/. 10.1136/jech-2012-201045 22766781

[6] Mendelsohn R, Emanuel K, Chonabayashi S, Bakkensen L (2012). The impact of climate change on global tropical cyclone damage. Nat. Clim. Change.

[7] Weinkle J, Maue R, Pielke R (2012). Historical global tropical cyclone landfalls. J. Clim..

[8] van Oldenborgh GJ (2017). Attribution of extreme rainfall from Hurricane Harvey, August 2017. Environ. Res. Lett..

[9] Domingues R (2021). Ocean conditions and the intensification of three major Atlantic Hurricanes in 2017. Mon. Weather Rev..

[10] Nguyen P (2014). Satellites track precipitation of super typhoon Haiyan. EOS Trans. Am. Geophys. Union.

[11] Wuebbles D (2014). CMIP5 climate model analyses: Climate extremes in the United States. Bull. Am. Meteor. Soc..

[12] USGCRP. *Climate Science Special Report: Fourth National Climate Assessment*, Volume I (eds Wuebbles, D. J. *et al.*) 10.7930/J0J964J6 (U.S. Global Change Research Program, 2017).

[13] Seneviratne, S. *et al.* Changes in climate extremes and their impacts on the natural physical environment. 109–230 (2012) 10.7916/D8-6NBT-S431.

[14] Musser JW, Watson KM, Gotvald AJ (2017). Characterization of peak streamflows and flood inundation at selected areas in North Carolina following Hurricane Matthew, October 2016. Open-File Rep..

[15] Easterling, D. R. *et al.* DigitalCommons@University of Nebraska-Lincoln Precipitation change in the United States.

[16] Lin Y, Zhao M, Zhang M (2015). Tropical cyclone rainfall area controlled by relative sea surface temperature. Nature Communications.

[17] Villarini G (2014). Sensitivity of tropical cyclone rainfall to idealized global-scale forcings. J. Clim..

[18] Patricola CM, Wehner MF (2018). Anthropogenic influences on major tropical cyclone events. Nature.

[19] Knutson T (2020). Tropical cyclones and climate change assessment: Part II: Projected response to anthropogenic warming. Bull. Am. Meteor. Soc..

[20] Sixth assessment report. https://www.ipcc.ch/report/ar6/wg1/.

[21] Risser MD, Wehner MF (2017). Attributable human-induced changes in the likelihood and magnitude of the observed extreme precipitation during Hurricane Harvey. Geophys. Res. Lett..

[22] Reed KA, Stansfield AM, Wehner MF, Zarzycki CM (2020). Forecasted attribution of the human influence on Hurricane Florence. Sci. Adv..

[23] Maxwell JT (2021). Recent increases in tropical cyclone precipitation extremes over the US east coast.. Proceedings of the National Academy of Sciences of the United States of America.

[24] Paerl HW (2019). Recent increase in catastrophic tropical cyclone flooding in coastal North Carolina, USA: Long-term observations suggest a regime shift. Sci. Rep..

[25] Touma D, Stevenson S, Camargo SJ, Horton DE, Diffenbaugh NS (2019). Variations in the intensity and spatial extent of tropical cyclone precipitation. Geophys. Res. Lett..

[26] Gao S, Mao J, Zhang W, Zhang F, Shen X (2021). Atmospheric moisture shapes increasing tropical cyclone precipitation in southern China over the past four decades. Environ. Res. Lett..

[27] Liu L, Wang Y (2020). Trends in landfalling tropical cyclone-induced precipitation over China. J. Clim..

[28] Chang CP, Yang YT, Kuo HC (2013). Large increasing trend of tropical cyclone rainfall in Taiwan and the roles of Terrain. J. Clim..

[29] Balaji M, Chakraborty A, Mandal M (2018). Changes in tropical cyclone activity in north Indian ocean during satellite era (1981–2014). Int. J. Climatol..

[30] Landsea CW, Harper BA, Hoarau K, Knaff JA (2006). Can we detect trends in extreme tropical cyclones?. Science.

[31] Kossin JP, Knapp KR, Vimont DJ, Murnane RJ, Harper BA (2007). A globally consistent reanalysis of hurricane variability and trends. Geophys. Res. Lett..

[32] Knutson TR (2010). Tropical cyclones and climate change. Nat. Geosci..

[33] Peterson TC (2014). Changes in weather and climate extremes: State of knowledge relevant to air and water quality in the United States. J. Air Waste Manage. Assoc.

[34] Gorooh VA, Asanjan AA, Nguyen P, Hsu K, Sorooshian S (2022). Deep neural network high SpatioTEmporal resolution precipitation estimation (Deep-STEP) using passive microwave and infrared data. J. Hydrometeorol..

[35] Gorooh VA (2020). Deep neural network cloud-type classification (DeepCTC) model and its application in evaluating PERSIANN-CCS. Remote Sens..

[36] Hayatbini N, Hsu KL, Sorooshian S, Zhang Y, Zhang F (2019). Effective cloud detection and segmentation using a gradient-based algorithm for satellite imagery: Application to improve PERSIANN-CCS. J. Hydrometeorol..

[37] Sadeghi M, Nguyen P, Hsu K, Sorooshian S (2020). Improving near real-time precipitation estimation using a U-Net convolutional neural network and geographical information. Environ. Model. Softw..

[38] Ashouri H (2015). PERSIANN-CDR: Daily precipitation climate data record from multisatellite observations for hydrological and climate studies. Bull. Am. Meteor. Soc..

[39] Sadeghi M (2021). PERSIANN-CCS-CDR, a 3-hourly 0.04° global precipitation climate data record for heavy precipitation studies. Sci. Data.

[40] Nguyen P (2020). PERSIANN dynamic infrared-rain rate model (PDIR) for high-resolution, real-time satellite precipitation estimation. Bull. Am. Meteor. Soc..

[41] Nguyen P (2020). PERSIANN dynamic infrared-rain rate (PDIR-Now): A near-real-time, quasi-global satellite precipitation dataset. J. Hydrometeorol..

[42] Sellars S (2013). Computational earth science: Big data transformed into insight. EOS Trans. Am. Geophys. Union.

[43] Sellars SL, Gao X, Sorooshian S (2015). An object-oriented approach to investigate impacts of climate oscillations on precipitation: A western United States case study. J. Hydrometeorol..

[44] Sellars SL, Kawzenuk B, Nguyen P, Ralph FM, Sorooshian S (2017). Genesis, pathways, and terminations of intense global water vapor transport in association with large-scale climate patterns. Geophys. Res. Lett..

[45] Shearer EJ (2020). Examination of global midlatitude atmospheric river lifecycles using an object-oriented methodology. J. Geophys. Res.: Atmos..

[46] Sadeghi M (2021). Application of remote sensing precipitation data and the CONNECT algorithm to investigate spatiotemporal variations of heavy precipitation: Case study of major floods across Iran (Spring 2019). J. Hydrol..

[47] Knapp KR, Kruk MC, Levinson DH, Diamond HJ, Neumann CJ (2010). The international best track archive for climate stewardship (IBTrACS): Unifying tropical cyclone data. Bull. Am. Meteor. Soc..

[48] Guzman O, Jiang H (2021). Global increase in tropical cyclone rain rate. Nat. Commun..

[49] Fowler HJ (2021). Anthropogenic intensification of short-duration rainfall extremes. Nat. Rev. Earth Environ..

[50] Kossin JP (2018). A global slowdown of tropical-cyclone translation speed. Nature.

[51] Li L, Chakraborty P (2020). Slower decay of landfalling hurricanes in a warming world. Nature.

[52] Lavender SL, McBride JL (2021). Global climatology of rainfall rates and lifetime accumulated rainfall in tropical cyclones: Influence of cyclone basin, cyclone intensity, and cyclone size. Int. J. Climatol..

[53] Zhao C (2018). Enlarging Rainfall Area of Tropical Cyclones by Atmospheric Aerosols. Geophys. Res. Lett..

[54] Traxl D, Boers N, Rheinwalt A, Bookhagen B (2021). The role of cyclonic activity in tropical temperature-rainfall scaling. Nat. Commun..

[55] Dhakal N, Tharu B (2018). Spatio-temporal trends in daily precipitation extremes and their connection with North Atlantic tropical cyclones for the southeastern United States. Int. J. Climatol..

[56] Kunkel KE (2010). Recent increases in U.S. heavy precipitation associated with tropical cyclones. Geophys. Res. Lett..

[57] Skok G, Bacmeister J, Tribbia J (2013). Analysis of tropical cyclone precipitation using an object-based algorithm. J. Clim..

[58] Climate at a Glance | National Centers for Environmental Information (NCEI). https://www.ncdc.noaa.gov/cag/.

[59] Gelaro R (2017). The modern-era retrospective analysis for research and applications, version 2 (MERRA-2). J. Clim..

[60] Adler RF (2018). The global precipitation climatology project (GPCP) monthly analysis (New version 2.3) and a review of 2017 global precipitation. Atmosphere.

[61] Saemian P (2021). Comprehensive evaluation of precipitation datasets over Iran. J. Hydrol..

[62] Huang W-R, Liu P-Y, Hsu J (2021). Multiple timescale assessment of wet season precipitation estimation over Taiwan using the PERSIANN family products. Int. J. Appl. Earth Obs. Geoinf..

[63] Nguyen P (2019). The CHRS data portal, an easily accessible public repository for PERSIANN global satellite precipitation data. Sci. Data.

[64] Nguyen P (2018). The PERSIANN family of global satellite precipitation data: A review and evaluation of products. Hydrol. Earth Syst. Sci..

[65] Daly C (2006). Guidelines for assessing the suitability of spatial climate data sets. Int. J. Climatol..

[66] Omranian E, Sharif HO, Tavakoly AA (2018). How well can global precipitation measurement (GPM) capture hurricanes? Case Study: Hurricane Harvey. Remote Sens..

[67] Prat OP, Nelson BR (2013). Precipitation contribution of tropical cyclones in the southeastern United States from 1998 to 2009 using TRMM satellite data. J. Clim..

[68] Larson J, Zhou Y, Higgins RW (2005). Characteristics of landfalling tropical cyclones in the United States and Mexico: Climatology and interannual variability. J. Clim..

[69] Lau KM, Zhou YP, Wu HT (2008). Have tropical cyclones been feeding more extreme rainfall?. J. Geophys. Res.: Atmos..

[70] Jiang H, Zipser EJ (2010). Contribution of tropical cyclones to the global precipitation from eight seasons of TRMM data: Regional, seasonal, and interannual variations. J. Clim..

[71] Schreck CJ, Molinari J (2011). Tropical cyclogenesis associated with kelvin waves and the Madden–Julian oscillation. Mon. Weather Rev..

[72] Chavas DR, Emanuel KA (2010). A QuikSCAT climatology of tropical cyclone size. Geophys. Res. Lett..

[73] Weber HC, Lok CCF, Davidson NE, Xiao Y (2014). Objective estimation of the radius of the outermost closed isobar in tropical cyclones. Trop. Cyclone Res. Rev..

[74] Dean L, Emanuel KA, Chavas DR (2009). On the size distribution of Atlantic tropical cyclones. Geophys. Res. Lett..

[75] Feldmann M, Emanuel K, Zhu L, Lohmann U (2019). Estimation of Atlantic tropical cyclone rainfall frequency in the United States. J. Appl. Meteorol. Climatol..

[76] Hampel, F. R. *Robust statistics: the approach based on influence functions*. (Wiley-Interscience; New York, 1986).

[77] Changnon SA, Kunkel KE (1995). Climate-related fluctuations in midwestern floods during 19211985. J. Water Resour. Plan. Manag..

[78] Dumouchel, W. Integrating a robust option into a multiple regression computing environment. In *Computer science and statistics: Proceedings of the 21st symposium on the interface* 297–302. (Alexandria: American Statistical Association, 1989).

