# Author Correction: Unveiling four decades of intensifying precipitation from tropical cyclones using satellite measurements

**DOI:** 10.1038/s41598-022-21626-1

**Published:** 2022-10-05

**Authors:** Eric J. Shearer, Vesta Afzali Gorooh, Phu Nguyen, Kuo‑Lin Hsu, Soroosh Sorooshian

**Affiliations:** 1 Center for Hydrometeorology and Remote Sensing (CHRS), Department of Civil and Environmental Engineering, University of California, Irvine, Irvine, CA USA; 2 Department of Earth System Science, University of California, Irvine, Irvine, CA USA

Correction to: *Scientific Reports* https://doi.org/10.1038/s41598-022-17640-y, Published online 09 August 2022

The original version of this Article contained errors in the Abstract and the Results.

In the Abstract,

“Increases in rainfall rates have boosted the mean precipitation volume of global TCs by 7–15%/year, with the starkest rises seen in the North Atlantic, South Indian, and South Pacific basins (maximum 59–64% over 40 years).”

now reads:

“Increases in rainfall rates have boosted the mean precipitation volume of global TCs by 7–15% over 40 years, with the starkest rises seen in the North Atlantic, South Indian, and South Pacific basins (maximum 59–64% over 40 years).”

In addition, in the Results section,

“The time series at the top-right of Fig. 2 provide a summarizing view of global ⟨*V*⟩ trends: increasing at a rate of 0.28 ± 0.10% per year or 7–18% over 40 years, though not without a strong oscillation in the time series.”

now reads:

“The time series at the top-right of Fig. 2 provide a summarizing view of global ⟨*V*⟩ trends: increasing at a rate of 0.28 ± 0.10% per year or 7–15% over 40 years, though not without a strong oscillation in the time series.”

The original Article has been corrected.

