# Supplementary material for: Unveiling four decades of intensifying precipitation from tropical cyclones using satellite measurements
Source: Sci Rep. 2022 Aug 9;12:13569. doi: 10.1038/s41598-022-17640-y (PMC9363467; doi:10.1038/s41598-022-17640-y)
Supplement: Supplementary file 1 — Supplementary Information. [file 41598_2022_17640_MOESM1_ESM.docx]

**Supplementary Information for**

Unveiling four decades of intensifying precipitation from tropical cyclones using satellite measurements

Eric J. Shearer, Vesta Afzali Gorooh, Phu Nguyen, Kuo-lin Hsu, Soroosh Sorooshian

Eric J. Shearer

Email: [eshearer@uci.edu](mailto:eshearer@uci.edu)

**This PDF file includes:**

Figure S1 to S3

**
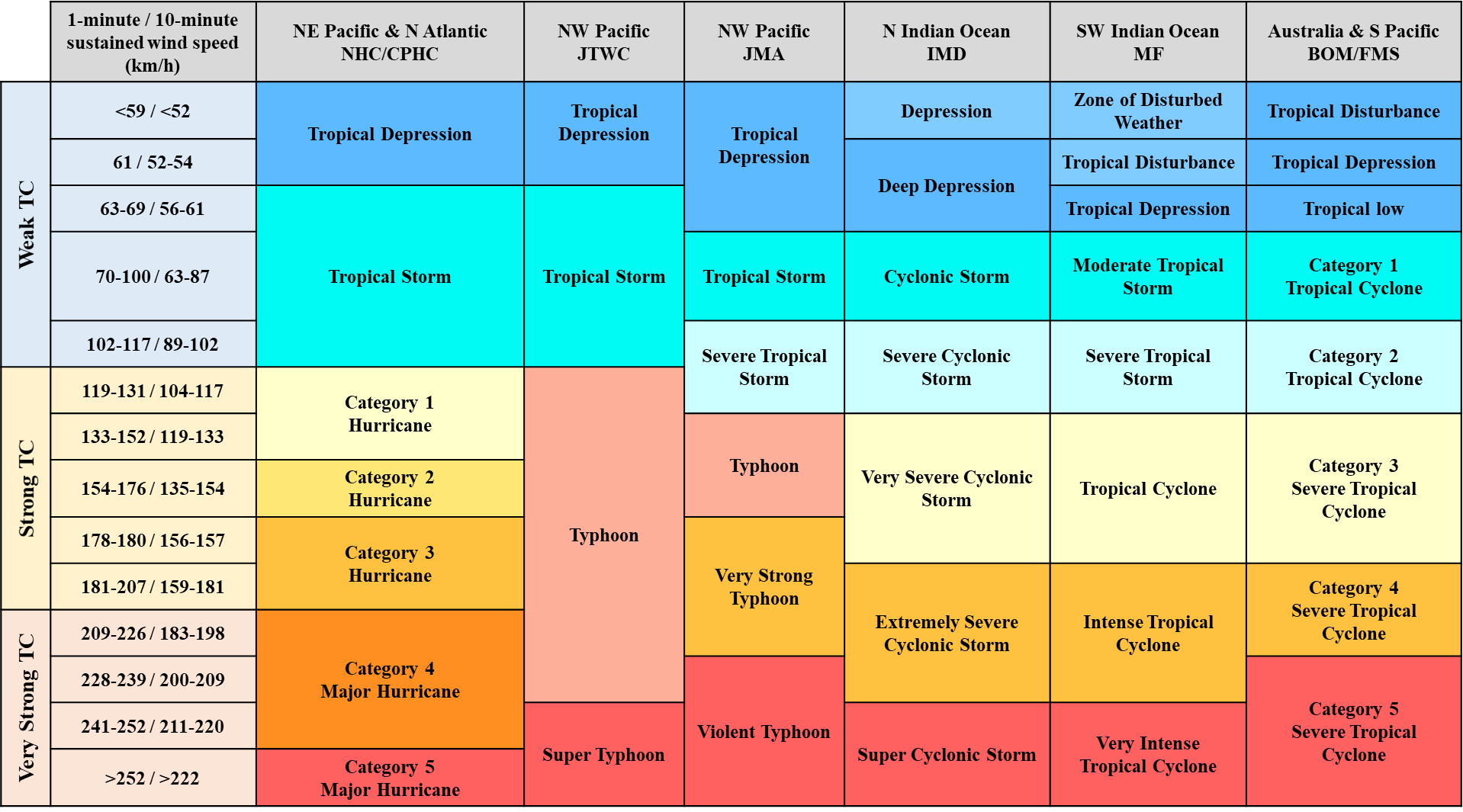
**

**Figure S1.** The grading scale used in this paper compared to 1-minute and 10-minute wind speed along with regional meteorological offices’ grading categories.


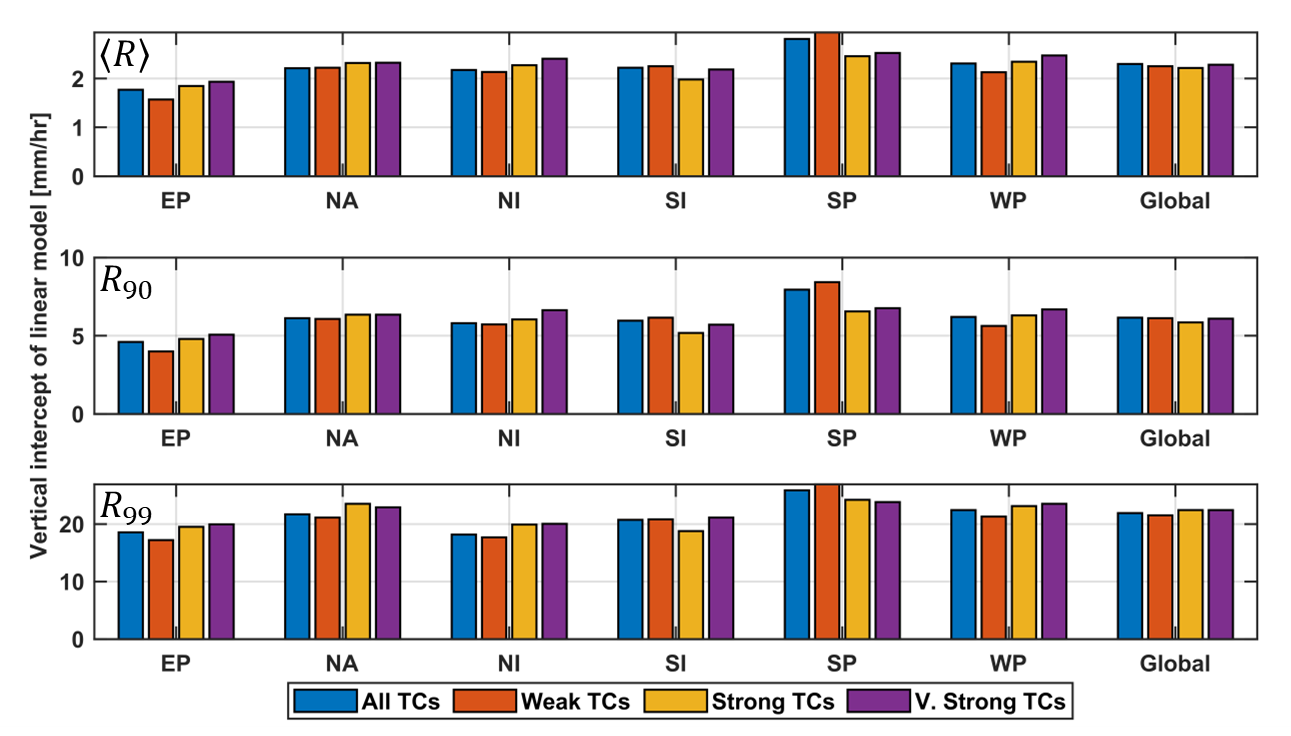


**Figure S2.** The values of the vertical intercepts for the linear models fit in Figure 1 for mean rainfall (top), the 90^th^ percentile of rainfall (middle), and the 99^th^ percentile of rainfall (bottom).


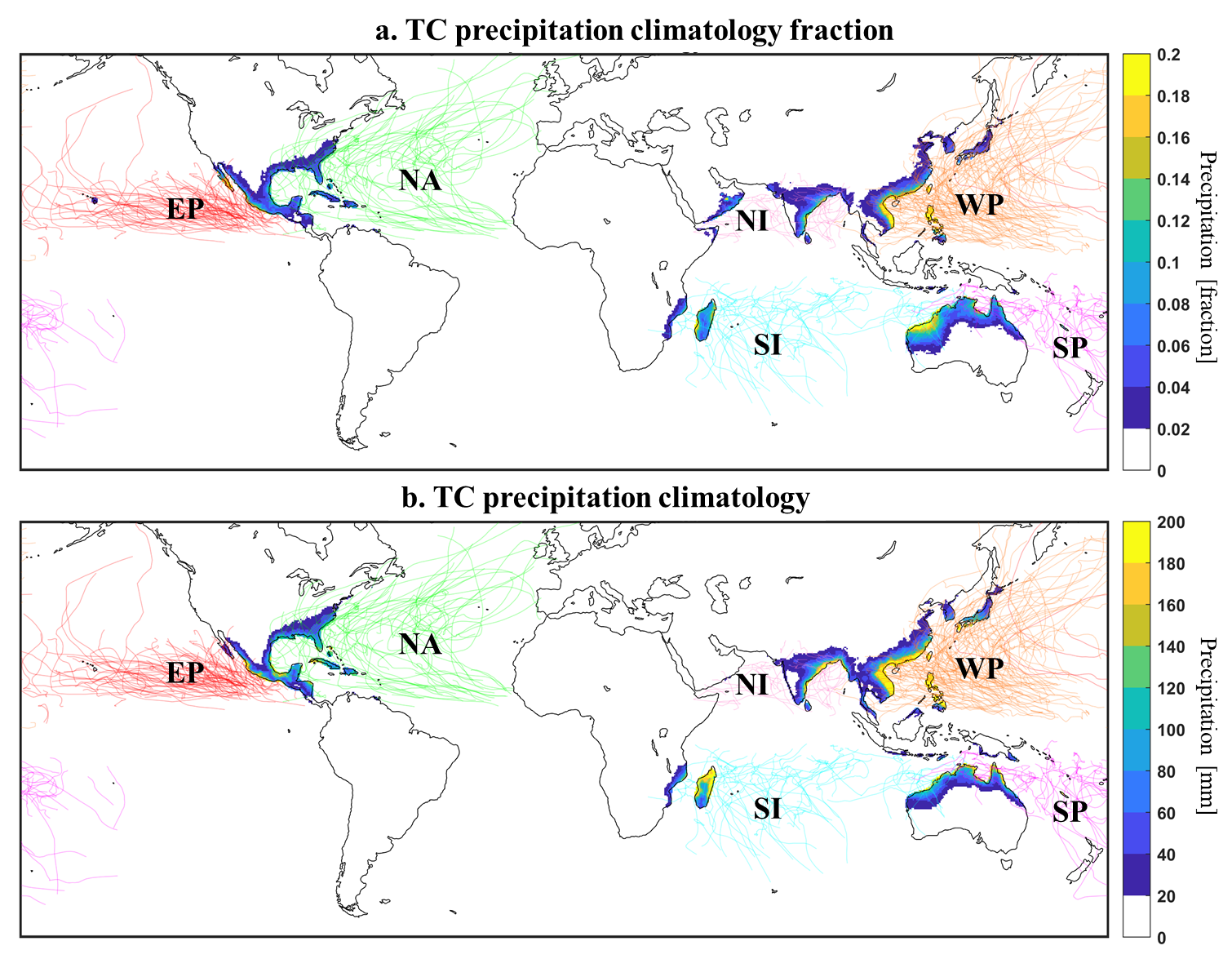


**Figure S3.** The distribution of landfalling TC precipitation over 1980-2019. a. TC precipitation climatology as a fraction of total precipitation climatology with color-coded lines indicating TC tracks over the 2015-2019 period. b. TC precipitation climatology in mm.
